# Deconvoluting the Electrophysiological Signatures of Myocardial Ischemia using a Validated Machine Learning Framework

**DOI:** 10.12688/f1000research.171338.2

**Published:** 2026-01-31

**Authors:** Ahmad Mahmood, Kiel Jacqueline, Joanne Lac

**Affiliations:** 1 Royal Free London NHS Foundation Trust, London, England, UK; 2 Boston University, Boston, Massachusetts, USA; 3 University College London, London, England, UK

**Keywords:** Myocardial ischemia; Machine learning; Electrophysiology; Cardiomyocyte; Action potential; Computational modeling

## Abstract

**Background:**

Myocardial ischemia is a dynamic, complex process characterized by hyperkalemia, acidosis, and ATP depletion. While these three conditions alter cardiomyocyte electrophysiology, it is difficult to discern how much each one individually contributes to the resulting changes in action potential (AP). In this study, we test whether machine learning can deconvolute these distinct ischemic patterns within a single AP.

**Methods:**

We developed a multi-target regression model trained on data generated by the Luo-Rudy (1991) computational model of a ventricular cardiomyocyte, simulating a wide range of ischemic conditions. The model was designed to predict two continuous variables: extracellular potassium concentration ([K ^+^]o) and intracellular pH (pHi).

**Results:**

The model achieved high accuracy on a held-out test set, with mean squared errors (MSE) below 0.25 for [K ^+^]o and below 0.01 for pHi. To further generalize this model, we applied this trained model to a structurally distinct model, the Ten Tusscher (2006) framework. We were able to accurately predict [K ^+^]o and pHi from APs, demonstrating that the learned principles are robust. A feature importance analysis revealed that resting membrane potential (RMP) was the strongest predictor for [K ^+^]o, while action potential duration (APD) is most important for predicting pHi, underscoring these distinct cardiomyocyte electrophysiological patterns

**Conclusions:**

Our approach can distinguish distinct ischemic drivers and has potential for *in silico* drug screening and mechanistic analysis.

## Author summary

During a myocardial infarction, or heart attack, cardiac myocytes experience an oxygen deficiency, increase in potassium outside the cell, and an increase in intracellular acid. These conditions contribute to an altered AP, however, it is difficult to discern the individual contributions of each condition. In this study, we explored whether a computer could learn to identify distinct signatures of damage. By simulating ischemia in a virtual heart cell, we generated thousands of APs using trusted computer models. With a trained machine learning system with high accuracy, we then analyzed the shape of these APs to predict the levels of extracellular potassium and intracellular acidity. To ensure the generalizability of our findings to further models, we tested our method on an entirely different, modern cardiac cell model. Our system maintained high accuracy despite its lack of familiarity with this model, supporting the robustness of our approach. Finally, we showed that the system could be used to evaluate how well a simulated drug protects heart cells, suggesting a new direction for testing therapies virtually.

## Introduction

Myocardial ischemia, a defining feature of heart attacks, initiates a series of interconnected pathological processes at the cellular level. As blood flow becomes restricted, oxygen supply rapidly diminishes, inhibiting aerobic metabolism and depleting ATP. This energy shortage leads to a cascade of disruptions in the cardiomyocyte’s internal environment, most notably, a rise in extracellular potassium concentration ([K ^+^]o) due to Na ^+^/K ^+^ pump failure, and a drop in intracellular pH (pHi) caused by lactate buildup through anaerobic glycolysis. ^1^

Together, these stressors, hyperkalemia, acidosis, and ATP depletion, profoundly alter the electrophysiological properties of cardiomyocytes. Hyperkalemia causes depolarization of the RMP, while acidosis interferes with the function of key ion channels, including the fast sodium current (INa) and the L-type calcium current (ICaL). At the same time, ATP depletion activates the ATP-sensitive potassium current (IK (ATP)), which significantly shortens the APD. ^2^ The resulting ischemic AP reflects a complex interplay of these pathophysiological changes.

Although the general shape of ischemic APs is well documented, characterized by depolarized RMP, slower upstroke, and shortened duration, it remains extremely difficult to determine which specific pathological factor is primarily responsible for these changes in a given case. For instance, two cardiomyocytes may display similarly short APDs due to different underlying combinations of hyperkalemia and acidosis. The ability to isolate and quantify the individual effects of these contributors could greatly improve our understanding of ischemic tissue states and support the development of more precisely targeted therapies.

Recent years have seen an increasing interest in using machine learning to identify cardiac pathology from electrophysiological signals. Clinical models have employed ECG-derived features, such as P wave dispersion and QRS duration, to detect obstructive coronary artery disease and stratify ischemic risk in high-risk cohorts. For example, Yilmaz et al. (2023) ^3^ used ECG features from treadmill stress tests to predict obstructive coronary artery disease with high accuracy. Similarly, Cicek et al. (2024) ^4^ developed a risk prediction model for perioperative myocardial injury in elderly surgical patients, integrating clinical and signal-derived data. These models underscore the clinical relevance and feasibility of ML-driven signal analysis in cardiology. However, these do not address direct mechanistic insight into the ionic or cellular substrates of the observed abnormalities. In contrast, our *in silico* modeling approach offers interpretable links between waveform changes and specific pathophysiological drivers, serving as a bridge between computational biology and clinical diagnostics.

In this study, we explore the potential of a machine learning approach, specifically a multi-target regression framework, to infer the distinct influences of hyperkalemia and acidosis from AP waveform features. We propose a three-phase strategy to investigate this hypothesis. First, we train Random Forest regressors on a synthetic dataset generated using the Luo-Rudy (1991) ^5^ computational model. Next, we test the generalizability of the trained models using an independent dataset created with the structurally distinct Ten Tusscher (2006) ^6^ model. Finally, we apply the validated framework to a simulated drug intervention in a severely ischemic cell to evaluate its potential use in quantifying therapeutic effects *in silico.*

## Materials and methods

### Computational models of the ventricular cardiomyocyte

This study utilized two widely accepted computational models of human ventricular cardiomyocytes.

1. Luo-Rudy (1991) Model (LRd): The main training dataset was generated using the LRd model, ^5^ a robust and extensively validated framework. Its reliability and stability across a broad range of physiological and pathological conditions made it ideal for producing a large-scale dataset for machine learning.
2. Ten Tusscher et al. (2006) Model (TT06): For validation purposes, we used the epicardial configuration of the TT06 model, ^6^ which offers a more recent and detailed representation of ventricular electrophysiology. It includes refined descriptions of calcium handling and a wider range of ionic currents. The structural differences between TT06 and LRd made it an effective choice to test the generalizability of our machine learning framework.

### Simulating ischemic conditions

To simulate ischemic conditions, we simultaneously varied three key physiological parameters in both models:

• Hyperkalemia: Extracellular potassium concentration ([K ^+^]o) was adjusted from a normal value of 5.4 mM to a maximum of 12.5 mM to represent severe ischemia.
• Acidosis: Intracellular pH (pHi) was decreased from a baseline of 7.4 to as low as 6.5. The impact of acidosis was modeled as a reduction in the maximum conductance of the fast sodium current (INa) and the slow inward/L-type calcium current (Isi/ICaL), consistent with experimental findings. ^7^
• ATP Depletion: Reduced ATP availability was mimicked by activating the ATP-sensitive potassium current (IK (ATP)). The conductance of this current (GK (ATP)) was increased from 0 (representing normal conditions) up to 0.3 mS/μF to simulate progressively severe ischemia.

### *In Silico* data generation

Using the LRd model, we generated a training dataset composed of 150 AP traces. These simulations were categorized into three groups: ‘Healthy,’ ‘Moderate Ischemia,’ and ‘Severe Ischemia.’ Parameter values were sampled from ranges defined in the accompanying Python script. Each simulation produced a set of electrophysiological features for model training.

Separately, we generated a validation dataset of 50 APs using the TT06 model. These were sampled from the ‘Moderate’ and ‘Severe Ischemia’ parameter ranges to evaluate the machine learning framework’s ability to generalize across models.

### Electrophysiological feature extraction

For every simulated AP, six key biomarkers were computed:

1. Resting Membrane Potential (RMP): The membrane potential just before stimulation.
2. Peak Potential (Peak V): The maximum voltage reached during the AP.
3. Action Potential Amplitude (APA): The difference between Peak V and RMP.
4. Maximum Upstroke Velocity (dV/dtmax): The steepest slope of the AP upstroke.
5. Action Potential Duration at 90% Repolarization (APD90).
6. Action Potential Duration at 50% Repolarization (APD50).

### Machine learning framework

All custom code used to generate simulations, extract electrophysiological features, and train the regression models is openly available on Zenodo. ^8^

**Figure 1.**
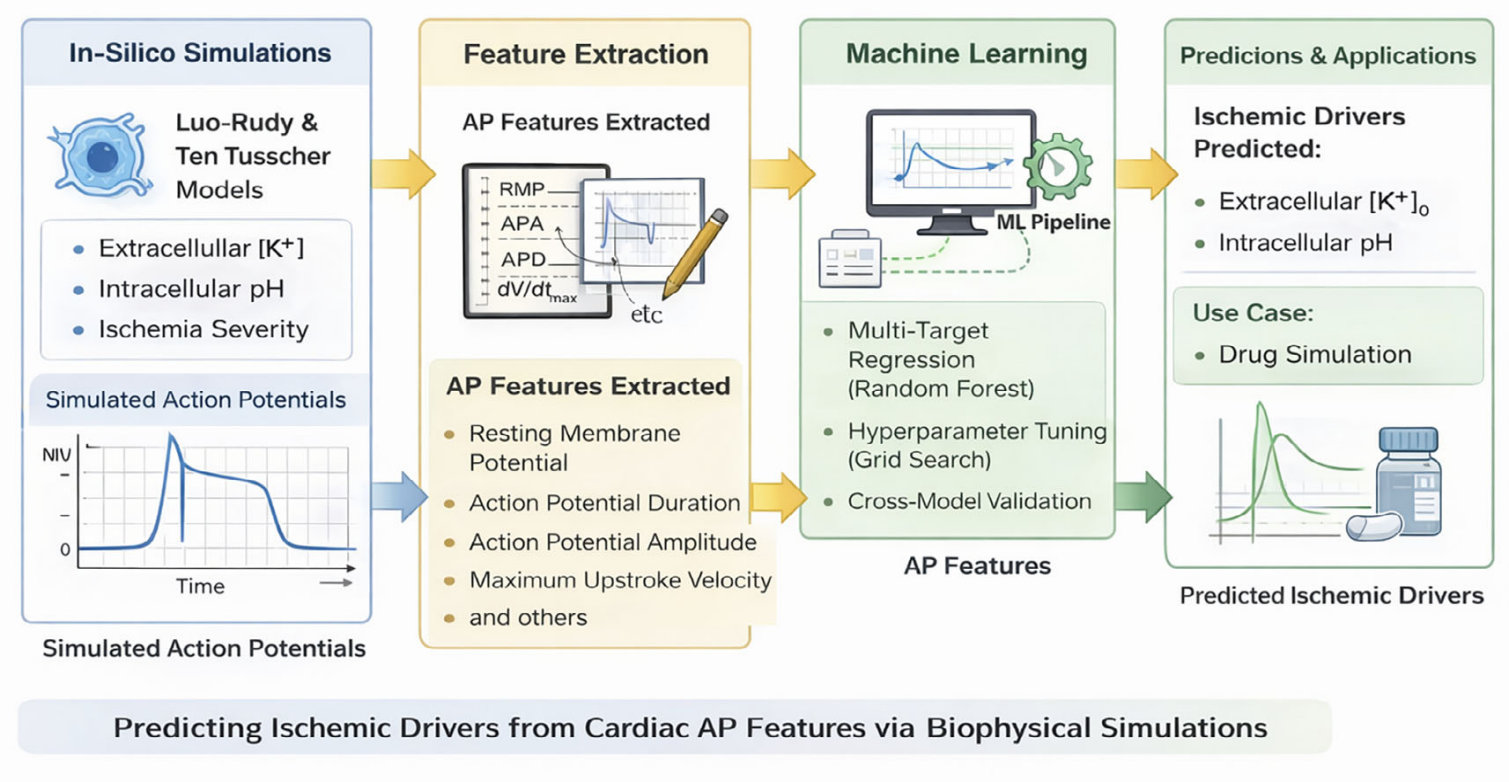
Overview of the in-silico modeling and machine learning pipeline. The workflow consists of four key stages: (1) simulation of cardiac action potentials under varying ischemic conditions using the Luo-Rudy and Ten Tusscher models, (2) extraction of electrophysiological features such as resting membrane potential, AP duration, amplitude, and upstroke velocity, (3) multi-target Random Forest regression with hyperparameter tuning to predict intracellular pH and extracellular potassium levels, and (4) model validation and application to a simulated drug scenario.

To support readers who may be less familiar with computational electrophysiology, we include a schematic ( Figure 1) summarizing the full pipeline, from biophysical simulations and feature extraction to machine learning and application.

Random Forest hyperparameters, including the number of estimators, maximum depth, and minimum samples per split, were tuned using a grid search with five-fold cross-validation on the training dataset. Performance was evaluated using mean absolute error across the two target variables (pHi and [K+]o), and the hyperparameter configuration yielding the lowest average error was selected for final model training.

Our analysis centered around a multi-target regression framework. The six extracted features served as input variables for two parallel Random Forest Regressor models implemented using scikit-learn in Python.

• Model 1 (K ^+^ Regressor): Trained to predict the continuous value of extracellular potassium ([K ^+^]o).
• Model 2 (pH Regressor): Trained to predict the continuous value of intracellular pH (pHi).

Each regressor consisted of an ensemble of 150 decision trees. Both models were trained exclusively on the dataset derived from the Luo-Rudy model.

### Cross-model validation protocol

The central evaluation of our framework involved cross-model validation. The regressors trained on the LRd dataset were used to estimate [K ^+^]o and pHi values from the APs in the TT06 validation dataset. Model performance was assessed by calculating the Mean Squared Error (MSE) between predicted and actual values, and by visually inspecting prediction plots.

### Simulated pharmacological rescue

To demonstrate a practical use case, we conducted a virtual pharmacological intervention. We selected one severely ischemic AP from the TT06 validation set. The trained models were first used to assess its baseline ischemic state by predicting [K ^+^]o and pHi. Next, a simulated drug was applied by setting the conductance of IK (ATP) to zero, mimicking a complete channel block. A new AP was then generated for the “treated” cell, and the updated features were input into the same regressors to quantify any change in the predicted pathological parameters.

### Code availability

The complete Python script used to generate all data, perform the analysis, and create the figures is openly available at Zenodo: https://doi.org/10.5281/zenodo.17216134.

The development repository is also accessible on GitHub: https://github.com/mahmood789/DIF.

## Results

### Ischemic severity alters AP morphology in a dose-dependent manner

Simulations using the LRd model revealed clear dose-dependent changes in AP waveforms as ischemia progressed. APs showed increasingly depolarized RMPs, reduced amplitude and upstroke velocity, and shorter APDs, all consistent with known electrophysiological effects of ischemia. ^1, 2^

### Machine learning models deconvolute ischemic factors

Both Random Forest regressors performed well on the LRd test set, accurately predicting [K ^+^]o and pHi. When applied to the independent TT06 dataset, the models retained strong predictive accuracy. MSE values were low, and predicted values tracked closely with actual parameters, demonstrating that the models had learned generalizable physiological relationships rather than model-specific patterns.

### Cross-model validation protocol to confirm generalizability

The central evaluation of our framework involved cross-model validation. The regressors trained on the LRd dataset were used to estimate [K ^+^]o and pHi values from the APs in the TT06 validation dataset. Model performance was assessed by calculating the Mean Squared Error (MSE) between predicted and actual values, and by visually inspecting prediction plots.

### Feature importance reveals distinct electrophysiological signatures

Analyzing feature importance revealed that [K ^+^]o predictions depended heavily on RMP, expected, given the Nernst relationship between potassium concentration and membrane potential. By contrast, pHi predictions drew primarily on APD90 and APA, reflecting acidosis’ broader effects on multiple ion channels. ^1, 7^

### Application: Quantifying the efficacy of a simulated pharmacological intervention

To demonstrate the framework’s practical utility, we selected a severely ischemic “patient” cell from the Ten Tusscher validation set. The model diagnosed this cell with elevated predicted [K ^+^]o and reduced pHi. We then simulated targeted drug intervention by blocking IK (ATP), which visibly improved the AP waveform, restoring a more hyperpolarized resting potential and prolonging its duration. When this treated AP was re-analyzed, the framework reflected a clear improvement in predicted ionic values, highlighting a partial rescue of the ischemic state. These results illustrate the model’s potential for *in silico* screening and quantitative evaluation of anti-ischemic therapies.

## Discussion

This study introduces a machine learning-based method for inferring specific ischemic stressors, hyperkalemia and acidosis, from single AP waveforms. Our results show that although these conditions co-occur and influence similar AP features, their electrophysiological “signatures” can be analyzed and separated by trained models. That said, the relatively modest dataset size (150 training APs and 50 validation APs) raises questions about robustness. Although the regressors performed well, expanding the training set or applying data augmentation strategies would strengthen confidence in model generalizability and reduce the risk of overfitting.

Crucially, the regressors trained on the Luo-Rudy model maintained high predictive accuracy when applied to the Ten Tusscher model. We selected Random Forest regressors for their interpretability and robustness with limited data. Nonetheless, alternative machine learning methods, such as gradient boosting or neural networks, could capture nonlinearities differently. Future work may compare performance across these approaches to identify optimal strategies for ischemic feature deconvolution. This cross-model validation provides strong evidence that the learned relationships reflect underlying principles of cardiac electrophysiology.

Feature importance analysis reinforced physiological expectations: RMP reflected extracellular potassium levels, ^1^ while APD and amplitude were indicative of pH-related effects. ^7^ The model’s successful use in simulating and quantifying a drug’s electrophysiological impact also points to its utility as a screening tool in computational pharmacology.

While clinical machine learning approaches using ECG data have shown significant promise in diagnostic and risk stratification tasks, they often provide limited insight into the underlying physiological mechanisms driving the observed signals. For example, Yilmaz et al. (2023) ^3^ used morphological features from P, QRS, and T waves during treadmill exercise testing to accurately predict obstructive coronary artery disease. Similarly, Cicek et al. (2024) ^4^ developed a risk prediction model for perioperative myocardial injury in elderly patients undergoing non-elective surgery, combining signal-derived and clinical variables. These studies demonstrate the growing clinical relevance of data-driven approaches to cardiac risk assessment. However, such models typically focus on statistical associations between features and outcomes, without directly modeling the underlying ionic or metabolic processes. In contrast, our *in silico* modeling framework explicitly simulates the biophysical effects of ischemic stressors, allowing for a transparent mapping between waveform changes and specific physiological drivers ([K ^+^]o and pHi). By learning from synthetic action potentials tied to known physiological conditions, our approach offers a novel, interpretable bridge between computational modeling and clinical diagnostics. This distinction situates our work not as a replacement for clinical machine learning tools, but as a complementary platform for hypothesis generation, virtual screening, and mechanistic insight.

Limitations of this study include the use of simplified cell models and the exclusion of other ischemic contributors like mechanical stretch, sympathetic stimulation, or reactive oxygen species. While our framework demonstrates strong predictive accuracy in silico, it has not yet been tested against experimental data. A clear next step is validation against electrophysiological recordings in ischemic cardiomyocytes, either through patch-clamp experiments or extracellular field potential measurements in animal ischemia models. Such experimental validation would not only strengthen biological plausibility but also provide critical insight into the translational robustness of the approach.

Additionally, although ATP-sensitive currents were incorporated through IK (ATP), the framework did not directly predict ATP depletion as an output. Extending the model to infer ATP levels would provide a more complete representation of ischemic pathophysiology, particularly in the context of metabolic stress. Future work should also explore tissue-level simulations and intercellular coupling to bridge the gap between single-cell models and translational applications.

Nonetheless, the present findings offer a strong foundation for data-driven approaches to studying and treating myocardial ischemia. In particular, future applications could move beyond single-cell models to incorporate tissue-level simulations, extracellular field potentials, or even clinical ECG recordings. Such extensions would help bridge the gap between mechanistic insight and translational utility in human patients.

## Ethics and consent

Ethical approval and consent were not required for this study.

## Data Availability

All data underlying the results are available from the Zenodo repository: https://doi.org/10.5281/zenodo.17216134. ^8^ The dataset includes simulated action potential traces, extracted electrophysiological features, and Python scripts used for model training and analysis.
- Values behind reported means and figures are provided in the dataset. - No participant data or personal identifiers were used. - Data are shared under a Creative Commons Zero v1.0 Universal

## Software availability

• Source code available from: https://github.com/mahmood789/DIF
• Archived source code available from: https://doi.org/10.5281/zenodo.17216134 ^8^
• License: **CC0-1.0 license**

### Extended data

Extended data files, including the full list of simulation parameters, feature extraction scripts, and validation datasets, are available on Zenodo: https://doi.org/10.5281/zenodo.17216134. ^8^

These materials are shared under the Creative Commons Zero v1.0 Universal.
