# Supplementary material for: Deconvoluting the Electrophysiological Signatures of Myocardial Ischemia using a Validated Machine Learning Framework
Source: F1000Res. 2026 Jan 31;14:1341. Originally published 2025 Dec 1. [Version 2] doi: 10.12688/f1000research.171338.2 (PMC12891950; doi:10.12688/f1000research.171338.2)
Supplement: Peer review history

# Reviewer report: Reviewer response for version 2

**DOI:** 10.5256/f1000research.195736.r454211

**Published:** 2026-02-10

**Authors:** Cenitta D

**Affiliations:** 1 Manipal Institute of Technology, Manipal Academy of Higher Education, Manipal, Karnataka, India

The revised version shows clear improvement and meets the requirements.

Is the work clearly and accurately presented and does it cite the current literature?

Partly

If applicable, is the statistical analysis and its interpretation appropriate?

Yes

Are all the source data underlying the results available to ensure full reproducibility?

Yes

Is the study design appropriate and is the work technically sound?

Yes

Are the conclusions drawn adequately supported by the results?

Yes

Are sufficient details of methods and analysis provided to allow replication by others?

Yes

Reviewer Expertise:

Machine learning, Heart disease prediction

I confirm that I have read this submission and believe that I have an appropriate level of expertise to confirm that it is of an acceptable scientific standard.

# Reviewer report: Reviewer response for version 2

**DOI:** 10.5256/f1000research.195736.r454212

**Published:** 2026-02-02

**Authors:** Mert İlker Hayıroğlu

**Affiliations:** 1 Dr. Siyami Ersek Thoracic and Cardiovascular Surgery Training and Research Hospital, Istanbul, Turkey

The manuscript is acceptable in its final status

Is the work clearly and accurately presented and does it cite the current literature?

Partly

If applicable, is the statistical analysis and its interpretation appropriate?

Yes

Are all the source data underlying the results available to ensure full reproducibility?

Yes

Is the study design appropriate and is the work technically sound?

Yes

Are the conclusions drawn adequately supported by the results?

Yes

Are sufficient details of methods and analysis provided to allow replication by others?

Yes

Reviewer Expertise:

cardiology

I confirm that I have read this submission and believe that I have an appropriate level of expertise to confirm that it is of an acceptable scientific standard.

# Reviewer report: Reviewer response for version 1

**DOI:** 10.5256/f1000research.188937.r438218

**Published:** 2026-01-10

**Authors:** Cenitta D

**Affiliations:** 1 Manipal Institute of Technology, Manipal Academy of Higher Education, Manipal, Karnataka, India

This study presents a machine learning–based framework to deconvolute key electrophysiological contributors to myocardial ischemia—specifically extracellular hyperkalemia and intracellular acidosis—from single cardiomyocyte action potential (AP) waveforms. Using simulated data generated from the Luo–Rudy (1991) ventricular cell model, the authors trained multi-target Random Forest regression models to predict extracellular potassium concentration ([K⁺]o) and intracellular pH (pHi). The robustness and generalizability of the framework were evaluated via cross-model validation using the structurally distinct Ten Tusscher (2006) model. Feature-importance analysis highlighted physiologically meaningful relationships, and a simulated pharmacological intervention was used to demonstrate potential translational utility. Overall, the work is technically sound, well structured, and reproducible.

 Major Comments

 1. Clarity of Presentation and Use of Current Literature (Partly)

 The manuscript is clearly written, logically organized, and presents results in a transparent and interpretable manner. The methodological workflow—from simulation to feature extraction, regression modeling, and validation—is easy to follow.

 However, the literature review and discussion would benefit from broader contextualization within recent machine learning applications in cardiology. While the foundational electrophysiology and computational modeling references are appropriate, the manuscript does not sufficiently engage with recent AI-driven studies on myocardial injury prediction, ischemia assessment, or ECG-based machine learning diagnostics. Incorporating recent clinical and signal-based ML studies would strengthen the manuscript’s relevance and better position the contribution within the current research landscape.

 Required revision:

- Expand the Introduction or Discussion to include recent machine learning studies related to myocardial ischemia, cardiac risk prediction, or electrophysiological signal analysis, particularly those using ECG or waveform-based features.
- Explicitly contrast the present mechanistic, in-silico approach with clinically oriented ML models to clarify novelty and scope.

2. Study Design and Technical Soundness (Yes)

 The study design is appropriate and well justified. The use of two distinct and well-established ventricular cardiomyocyte models for training and cross-validation is a notable strength. The choice of Random Forest regression is suitable given the dataset size, the nonlinear relationships involved, and the emphasis on interpretability.

 The cross-model validation strategy convincingly demonstrates that the learned relationships reflect underlying electrophysiological principles rather than model-specific artifacts.

 3. Methods and Reproducibility (Yes)

 The manuscript provides sufficient methodological detail to enable replication. Model parameters, simulation ranges, extracted electrophysiological features, and evaluation metrics are clearly described. Importantly, the authors have made all source code, simulated datasets, and extended data publicly available, which fully supports transparency and reproducibility.

 4. Statistical Analysis (Yes)

 The statistical analysis is appropriate for the study objectives. The use of mean squared error for continuous regression targets is suitable, and interpretation of results is consistent with the reported metrics. Feature-importance analysis is used appropriately to support physiological interpretability rather than overstated causal claims.

 5. Data Availability and Reproducibility (Yes)

 All underlying data and code are openly accessible via Zenodo and GitHub, including raw simulation outputs and values behind figures. This fully satisfies reproducibility requirements.

 6. Validity of Conclusions (Yes)

 The conclusions are well supported by the results. Claims are appropriately scoped to in-silico modeling, and limitations—such as dataset size, simplified cellular models, and lack of experimental validation—are openly acknowledged. The discussion avoids overgeneralization to clinical settings, which is commendable.

 Minor Suggestions (Optional Improvements)

- Clarify whether hyperparameter tuning was performed for the Random Forest models and, if so, how it was conducted.
- Consider adding a brief schematic summarizing the end-to-end pipeline for readers less familiar with computational electrophysiology.

Overall Recommendation

 This is a technically strong and reproducible study that makes a meaningful methodological contribution to computational cardiology and electrophysiological modeling. The primary issue to be addressed is the limited engagement with recent machine learning literature in myocardial ischemia and cardiac diagnostics. Addressing this point would significantly enhance the manuscript’s contextual depth and appeal to a broader readership.

 Once this revision is made, the article will meet the standards of scientific soundness and relevance expected for indexing.

Is the work clearly and accurately presented and does it cite the current literature?

Partly

If applicable, is the statistical analysis and its interpretation appropriate?

Yes

Are all the source data underlying the results available to ensure full reproducibility?

Yes

Is the study design appropriate and is the work technically sound?

Yes

Are the conclusions drawn adequately supported by the results?

Yes

Are sufficient details of methods and analysis provided to allow replication by others?

Yes

Reviewer Expertise:

Machine learning, Heart disease prediction

I confirm that I have read this submission and believe that I have an appropriate level of expertise to confirm that it is of an acceptable scientific standard, however I have significant reservations, as outlined above.

# Response

**Published:** 2026-01-24

**Authors:** Jacqueline Kiel

**Affiliations:** Boston University College of Health and Rehabilitation Sciences Sargent College, Boston, Massachusetts, USA

We thank the reviewer for their thoughtful and constructive feedback. In response to the suggestion to broaden the discussion of current machine learning literature in cardiac diagnostics, we have revised the manuscript to incorporate and contextualize the two cited studies:

- *Yilmaz et al. (2023)* on ECG-based prediction of obstructive coronary artery disease using features from treadmill stress tests, and
- *Cicek et al. (2024)* on a clinical risk model for perioperative myocardial injury in elderly patients.

These works are now discussed in the Introduction and Discussion sections to emphasize the expanding clinical relevance of data-driven cardiac diagnostics. In the revised Discussion, we also clarify how our in-silico modeling framework complements such signal-based models by offering a physiologically grounded, interpretable approach that links specific waveform features to biophysical drivers (e.g., [K⁺]ₒ and *pHi*).

 We believe this enhancement significantly strengthens the manuscript's connection to the broader landscape of AI in cardiology and addresses the reviewer's concern regarding contextual depth.

 Additionally, to support readers less familiar with computational electrophysiology, we have included a schematic (Figure 1) summarizing the full modeling pipeline; from simulation and feature extraction through to machine learning and application. We believe this visual overview improves the manuscript’s accessibility and responds directly to the reviewer’s helpful recommendation.

 Thank you again for your valuable input, which has helped improve the clarity, accessibility, and clinical relevance of our work.

# Reviewer report: Reviewer response for version 1

**DOI:** 10.5256/f1000research.188937.r438213

**Published:** 2025-12-30

**Authors:** Mert İlker Hayıroğlu

**Affiliations:** 1 Dr. Siyami Ersek Thoracic and Cardiovascular Surgery Training and Research Hospital, Istanbul, Turkey

The manuscript presents a novel and technically sound approach to distinguishing the specific contributions of hyperkalemia and acidosis to action potential morphology using a multi-target regression model, with a particularly impressive cross-model validation strategy. However, the discussion regarding the broader clinical applicability and the current landscape of machine learning in predicting myocardial injury and coronary disease could be significantly enriched. To better contextualize the study within recent advancements in AI-driven cardiac risk assessment and signal analysis, the authors are encouraged to reference and discuss recent relevant works such as 'A New Risk Prediction Model for the Assessment of Myocardial Injury in Elderly Patients Undergoing Non-Elective Surgery' and 'Machine Learning Approach on High Risk Treadmill Exercise Test to Predict Obstructive Coronary Artery Disease by using P, QRS, and T waves' Features'. Incorporating these studies would highlight the expanding role of machine learning in both electrophysiological modeling and clinical diagnostics, thereby strengthening the manuscript's relevance to a wider audience

Is the work clearly and accurately presented and does it cite the current literature?

Partly

If applicable, is the statistical analysis and its interpretation appropriate?

Yes

Are all the source data underlying the results available to ensure full reproducibility?

Yes

Is the study design appropriate and is the work technically sound?

Yes

Are the conclusions drawn adequately supported by the results?

Yes

Are sufficient details of methods and analysis provided to allow replication by others?

Yes

Reviewer Expertise:

cardiology

I confirm that I have read this submission and believe that I have an appropriate level of expertise to confirm that it is of an acceptable scientific standard, however I have significant reservations, as outlined above.

## References

[1] : Machine Learning Approach on High Risk Treadmill Exercise Test to Predict Obstructive Coronary Artery Disease by using P, QRS, and T waves’ Features. *Current Problems in Cardiology* .2023;48(2) : 10.1016/j.cpcardiol.2022.101482 10.1016/j.cpcardiol.2022.101482 36336117

[2] : A New Risk Prediction Model for the Assessment of Myocardial Injury in Elderly Patients Undergoing Non-Elective Surgery. *Journal of Cardiovascular Development and Disease* .2024;12(1) : 10.3390/jcdd12010006 10.3390/jcdd12010006 39852284 PMC11765956

# Response

**Published:** 2026-01-24

**Authors:** Jacqueline Kiel

**Affiliations:** Boston University College of Health and Rehabilitation Sciences Sargent College, Boston, Massachusetts, USA

We sincerely thank the reviewer for their positive evaluation of the technical contribution of our study and for highlighting the importance of broader clinical contextualization.

 In response to the reviewer’s recommendation, we have revised both the Introduction and Discussion to incorporate recent developments in machine learning applied to cardiac diagnostics. Specifically, we now reference and discuss two suggested studies:

- Yilmaz et al. (2023)
- Cicek et al. (2024)

These additions help to position our mechanistic modeling framework within the broader landscape of AI-driven cardiac risk assessment. We emphasize how our *in silico* approach complements clinical ML models by providing interpretable, physiology-based insights into ischemic waveform changes; specifically the contributions of [K⁺]ₒ and pHi, which are not easily accessible through observational data alone.

 Additionally, we have included a schematic diagram (Figure 1) summarizing our end-to-end pipeline, from biophysical simulation through feature extraction and multi-target regression, to validation and application. We hope this will aid readers less familiar with computational electrophysiology in understanding the structure and goals of the study. We are grateful for the reviewer’s thoughtful input, which helped us significantly strengthen the clarity, clinical relevance, and accessibility of the manuscript.
